# Effects of rest interval and training intensity on jumping performance: a systematic review and meta-analysis investigating post-activation performance enhancement

**DOI:** 10.3389/fphys.2023.1202789

**Published:** 2023-06-23

**Authors:** Yiyan Chen, Qing Su, Juan Yang, Gen Li, Shiyan Zhang, Yuanyuan Lv, Laikang Yu

**Affiliations:** ^1^ Key Laboratory of Physical Fitness and Exercise, Ministry of Education, Beijing Sport University, Beijing, China; ^2^ Department of Sports Performance, Beijing Sport University, Beijing, China; ^3^ Ersha Sports Training Center of Guangdong Province, Guangzhou, China; ^4^ School of Physical Education and Sports Science, South China Normal University, Guangzhou, China; ^5^ China Institute of Sport and Health Science, Beijing Sport University, Beijing, China

**Keywords:** barbell squat, rest interval, training intensity, jumping performance, post-activation performance enhancement

## Abstract

**Background:** There is no clear agreement regarding the ideal rest interval and training intensity to optimize post-activation performance enhancement (PAPE) after barbell squat (BS). Therefore, the aim of this study was to investigate the effects of rest interval and training intensity on jumping performance due to PAPE.

**Methods:** Searches were performed in PubMed, Web of Science, and EBSCO databases. We included studies that satisfied the following criteria: (1) eligible studies should be randomized controlled trials; (2) eligible studies should investigate the acute effect of barbell squat-induced PAPE on jump performance; (3) eligible studies should use countermovement jump, squat jump, or vertical jump as the outcome measure.

**Results:** From 2518 search records initially identified, 19 studies were eligible for meta-analysis. Our meta-analysis results showed that BS had no significant effect on improving jumping performance due to PAPE (Cohen’s *d* = 0.09, *p* = 0.08). Subgroup analysis showed that rest interval between 0–1 min was detrimental to jumping performance (Cohen’s *d* = −0.33, *p* < 0.01), while rest intervals between 4–7 min (Cohen’s *d* = 0.31, *p* < 0.01) and 8-9 min (Cohen’s *d* = 0.26, *p* = 0.02) provided favorable jumping performance outcomes. In addition, low-intensity and moderate-intensity BS had no significant effect on jump performance, while high-intensity BS showed results consistent with rest interval.

**Conclusion:** Our study indicated that both low-intensity and moderate-intensity BS failed to induce PAPE, and it is recommended that future studies use high-intensity BS to induce PAPE. A rest interval of 4–9 min had a beneficial impact on jump height, and an interval range of 4–7 min seems to be the best rest interval between conditioning activity and jumping performance.

## Introduction

Among all types of warm-up strategies, post-activation performance enhancement (PAPE) is widely used by coaches (Docarmo et al., 2021). Due to the previous history of high-intensity muscle contractile in PAPE, it will generate acutely large power (GoŁAŚ et al., 2017; Sale, 2002). A previous study showed that a complex training protocol would be the available method to induce PAPE and to improve jumping performance (Poulos et al., 2018). Therefore, PAPE can be used as a warm-up and re-warm-up protocol not only in daily training but also before or during competitions (Maclntosh et al., 2012).

Previous studies have demonstrated that PAPE causes fatigue and potentiation in participants (Boullosa et al., 2011; Andrews et al., 2016; Masiulis et al., 2018), with fatigue or potentiation dominating at different rest intervals, which determines whether the exercise performance is improved (Koziris, 2012). Therefore, to better understand the relationship between fatigue and potentiation, it is crucial to strictly control the implementation of PAPE. There are many factors that can influence the effect of PAPE, for example, the rest interval between conditioning and main activity, training intensity, and the training level of athlete. However, the optimal rest interval between conditioning and primary activity has not been revealed. Previous studies have shown that PAPE could not be induced after conditioning activities and even found a decrease in performance, especially in the minute after conditioning activities (Kilduff et al., 2008; Lowery et al., 2012; Mola et al., 2014; Seitz et al., 2014; Sharma et al., 2018). In addition, significant changes in jumping performance were not observed some studies where the rest interval ranged from immediately to 15 min after the conditioning activities (Crum et al., 2012; da Silva Santos et al., 2016; Moir et al., 2011; Naclerio et al., 2015; Weber et al., 2008), which was most likely due to differences in training intensity. Previous studies have shown that moderate-intensity conditioning activities may better induce PAPE (Wilson et al., 2013; Dobbs et al., 2019; Garbisu-Hualde and Santos-Concejero, 2021), but there are no studies on the rest interval at different intensities.

Therefore, we conducted a systematic review and meta-analysis to assess the effects of rest intervals between conditioning and main activity and training intensity on jumping performance due to PAPE, to discuss the recommended intervals to be used when using different intensities, and to make recommendations to improve the ability to implement PAPE in practical situations.

## Methods

### Design

This systematic review and meta-analysis was conducted according to the criteria and recommendations of the Preferred Reporting Items for Systematic Reviews and Meta-Analyses (PRISMA 2020) (Page et al., 2021). The protocol for this systematic review and meta-analysis has been registered on PROSPERO (CRD42022339144).

### Search strategy

All the studies on the effects of PAPE on jumping performance before 26 May 2023 were searched in Pubmed, Web of Science, and EBSCO electronic databases, using the following MESH terms and text words: post-activation potentiation, PAP, post-activation performance enhancement, PAPE, jump, explosive power, rest interval, intensity. We also hand-searched reference lists of all correlational studies. All studies used for meta-analysis need to meet the following criteria: eligible studies should (1) be randomized trials (RCTs); (2) investigate the acute effect of barbell squat-induced PAPE on jump performance; (3) use countermovement jump (CMJ), squat jump (SJ), or vertical jump (VJ) as the outcome measure. Exclusion criteria were as follows: (1) Non-English language publications; (2) reviews and conference articles; (3) animal model publications.

### Data extraction and quality assessment

The documental information of all qualified studies includes author information, participant characteristics (*n*, age, gender), volume or intensity of conditioning activity, rest interval between conditioning and main activity, and jump height [mean and standard deviation (SD)]. Rest interval analysis included 7 arbitrary different subgroups in which the effects of the ranges from 0–1, 2–3, 4–7, 8–9, 10–11, 12–15, and 16 min and above were independently examined. Two reviewers independently reviewed the titles, abstracts, and full texts of all citations to identify studies reporting the effects of conditioning activity on jumping performance due to PAPE. The selection procedure was conducted according to inclusion and exclusion criteria. In case of any discrepancies between the two authors, a third author was involved in the discussion until a consensus was made.

The Cochrane collaboration tool, which includes items on selection bias, performance bias, detection bias, attrition bias, and reporting bias, was used to evaluate the quality of eligible studies. Each item was judged as either “low risk,” “unclear risk,” or “high risk” based on responses to the signaling questions, to make an overall bias judgment for the specific study outcome being assessed. The methodological quality of the included studies was judged based on the final summary (Higgins and Green, 2008; Tao et al., 2023).

### Data synthesis and analysis

As the included studies tended to report jumping performance outcomes for multiple rest intervals, we cannot assume that the results of rest intervals are independent and estimate the same results, statistical analyses were based on a three-level restricted maximum likelihood random effects model, using the “metafor”, for R package (Viechtbauer, 2010; Li et al., 2023). Using the computational approach described in the study by Assink and Wibbelink, (2016). The model illustrates the dependence of within-study effect sizes by providing within-study (level 2) and between-study (level 3) variance estimates. The primary outcome indicator included in this study was expressed as “mean ± SD”, using Cohen’s *d* to standardize the difference in change from baseline to post-intervention between the exercise and control groups. A positive Cohen’s *d* indicates an increase in jumping performance in the after intervention compared to before intervention. Total effect size (ES) values were assessed according to the Cohen’s *d* classification (*d* = 0.2–0.5, small; *d* = 0.5–0.8, medium; *d* = 0.8, large) (Higgins and Green, 2008). Heterogeneity was assessed by *I* ^2^ static. *I* ^2^ < 25% indicates no significant heterogeneity; 25% < *I* ^2^ < 50%, low heterogeneity; 50% < *I* ^2^ < 75%, medium heterogeneity; *I* ^2^ > 75%, high heterogeneity (Li et al., 2022; Zhen et al., 2022).

In subgroup analyses, we tried to use rest interval (0–1, 2–3, 4–7, 8–9, 10–11, 12–15, and 16 min and above), training intensity (low-intensity, moderate-intensity, and high-intensity), and rest intervals at different intensities (0–1, 2–3, 4–7, 8–9, 10–11, 12–15, and 16 min and above at low-intensity, moderate-intensity, and high-intensity, respectively) to investigate the effects of rest intervals between conditioning and primary activity and training intensity on jump performance due to PAPE. All analyses were performed using R4.2.2 (R Foundation for Statistical Computing, Vienna, Austria) (Team, 2011).

## Results

### Study selection

**FIGURE 1:**
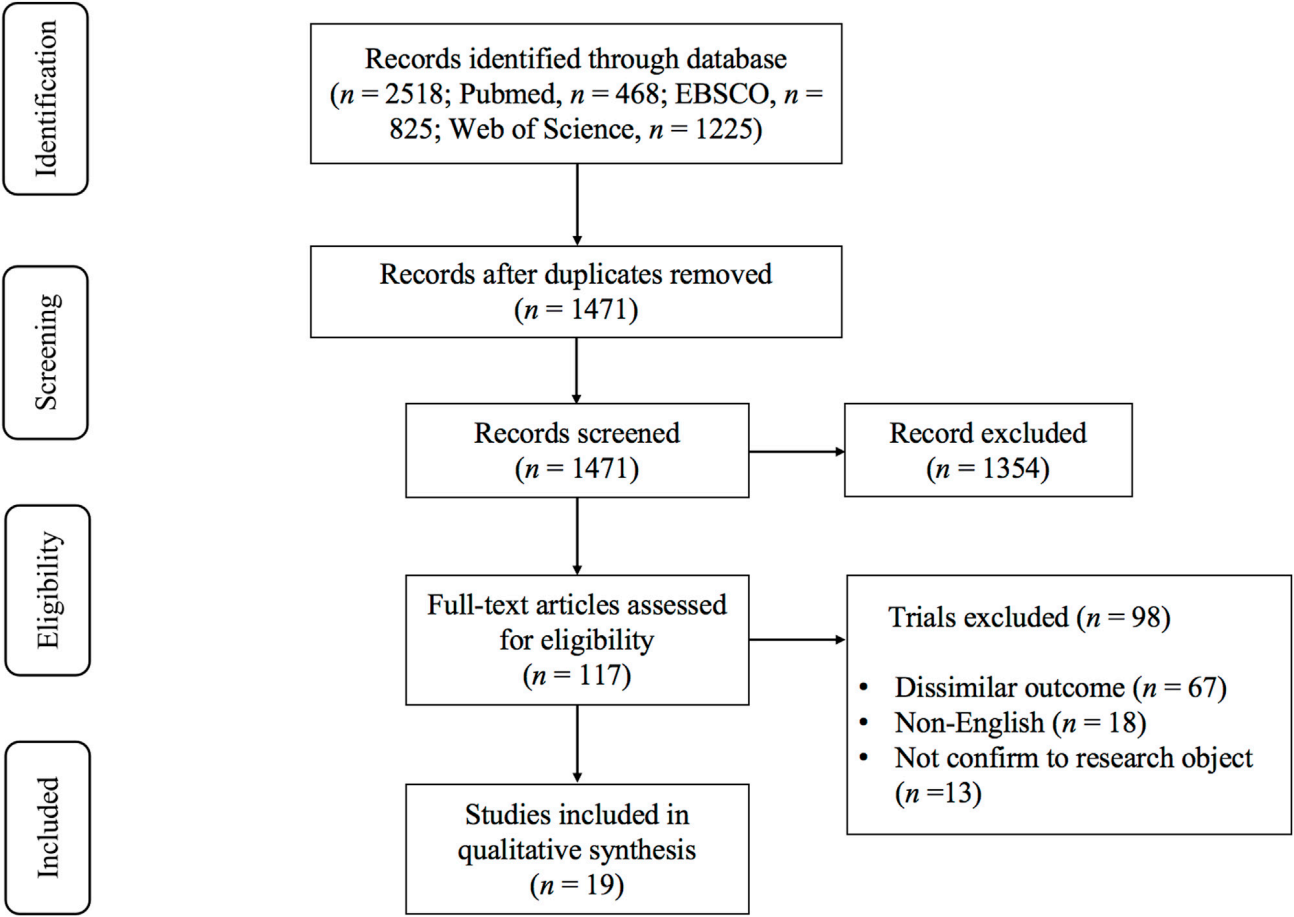
PRISMA flowchart of study selection.

The literature search results and searching procedure were shown in Figure 1. Among the 2518 articles identified, after reading the titles and abstracts, and then reading the full texts, 19 studies were considered eligible for meta-analysis (Atalag et al., 2021; Crum et al., 2012; da Silva Santos et al., 2016; Escobar Hincapié et al., 2021; Hirayama, 2014; Kilduff et al., 2008; Kilduff et al., 2011; Krcmar et al., 2021; Lowery et al., 2012; McCann and Flanagan, 2010; Mola et al., 2014; Moir et al., 2011; Naclerio et al., 2015; Rixon et al., 2007; Seitz et al., 2014; Sharma et al., 2018; Villalon-Gasch et al., 2020; Weber et al., 2008; Yi et al., 2022).

### Description of the included studies

The main characteristics of participants and training interventions were shown in Table 1. The included studies involved 255 participants. The average age of the participants was from 18 to 33 years old. One study involved untrained adults and 18 studies involved participants with training experience. According to the position statement of training intensity, we adjusted the intensity classification of conditioning activity according to the included research situation: the intensity of resistance training can be determined by the number of repetitions of the maximum weight at a time, and all included studies subjects were tested with one-repetition maximum (1RM) to determine the weight and number of times a barbell squat was used. And the intensity of the included literature was given directly in the article. Wilson et al. (2013) pointed out that the division of intensity can be derived by calculating power, so the method of dividing intensities in this paper also uses this method. Among the included studies, 13 studies conducted high-intensity BS (Atalag et al., 2021; da Silva Santos et al., 2016; Hirayama, 2014; Kilduff et al., 2008; Kilduff et al., 2011; Krcmar et al., 2021; Lowery et al., 2012; McCann and Flanagan, 2010; Mola et al., 2014; Moir et al., 2011; Rixon et al., 2007; Seitz et al., 2014; Sharma et al., 2018; Villalon-Gasch et al., 2020; Weber et al., 2008), 6 studies conducted moderate-intensity BS (Crum et al., 2012; Lowery et al., 2012; Hirayama, 2014; Naclerio et al., 2015; Escobar Hincapié et al., 2021; Yi et al., 2022), and 5 studies conducted low-intensity BS (Crum et al., 2012; da Silva Santos et al., 2016; Lowery et al., 2012; Moir et al., 2011; Hirayama, 2014). The rest interval between conditioning and main activity ranged from 0–24 min. Regarding outcome measures, 12 studies used CMJ as the outcome measure (da Silva Santos et al., 2016; Escobar Hincapié et al., 2021; Kilduff et al., 2008; Kilduff et al., 2011; Krcmar et al., 2021; Moir et al., 2011; Mola et al., 2014; Naclerio et al., 2015; Rixon et al., 2007; Sharma et al., 2018; Villalon-Gasch et al., 2020; Yi et al., 2022), 5 studies used VJ as the outcome measure (Atalag et al., 2021; Crum et al., 2012; da Silva Santos et al., 2016; Lowery et al., 2012; Hirayama, 2014), and 3 studies used SJ as the outcome measure (Krcmar et al., 2021; Seitz et al., 2014; Weber et al., 2008).

**TABLE 1:** Basic characteristics of studies included in the meta-analysis.

Study	Participants	Sample	Age	Type of CA	Sets/Repetitions/Intensity	Interval between CA	Jumping results
Seitz et al. (2014)	Rugby players	18F	18.8 ± 0.9	Squat	1/3/90% 1RM	15 s and 3, 6, 9, 12 min	SJ: 15 s ↓
							3, 6, 9, 12 min ↑
Villalon-Gasch et al. (2020)	Trained volleyball players	6F	22.18 ± 3.37	Half squat	1/5/50% 1RM, 1/3/90%1RM	8 min	CMJ: 8 min ↑
Mola et al. (2014)	Soccer players	11M	23 ± 4.5	Squat	1/3/75% 1RM, 1/2/90% 1RM, 1/1/100% 1RM	15 s and 4, 8, 12, 20 min	CMJ: 15 s ↓↓ 4 min ↑
							8 min ↓↓ 12 min ↓↓
							16 min ↑ 20 min ↑
Kilduff et al. (2008)	Trained rugby players	20M	25.4 ± 4.8	Squat	3/3/87% 1RM	15 s and 4, 8, 12, 16, 20, 24 min	CMJ: 15 s ↓↓ 4 min ↑
							8 min ↑↑ 12 min ↑
							16 min ↓ 20 min ↓
							24 min ↓
da Silva Santos et al. (2016)	Black-belt taekwondo athletes	9M	20.3 ± 5.2	Half squat	LL: 1/3/50% 1RM	10 min	CMJ: ND
					LH: 1/3/90% 1RM		
					HL: 3/3/50% 1RM		
					HH: 3/3/90% 1RM		
Naclerio et al. (2015)	Trained students	7M, 4F	25.4 ± 2.1	Parallel back squat	LV: 1/1/80% 1RM	15 s and 1, 2, 3, 5, 8, 12 min	CMJ: ND
					MV: 1/3/80% 1RM		
					HV: 2/3/80% 1RM		
Lowery et al. (2012)	Trained men	13M	21 ± 3	Squat	Low-intensity: 1/5/56% 1RM	0, 2, 4, 8, 12 min	VJ: ND
					Moderate-intensity: 1/4/70% 1RM	0, 2, 4, 8, 12 min	VJ: 0 min ↓↓ 4 min ↑↑
					High-intensity: 1/3/93% 1RM	0, 2, 4, 8, 12 min	VJ: 0 min ↓↓ 4 min ↑↑ 8 min ↑↑
McCann and Flanagan, (2010)	Volleyball athletes	7M, 7F	M: 20.86 ± 1.77 F: 19.14 ± 0.38	Back squat	1/5/5RM	4, 5 min	VJ: 4 min ↑↑
Kilduff et al. (2011)	Sprint swimmers	7M, 2F	22 ± 2	Back squat	1/3/87% 1RM	4, 8, 12, 16 min	CMJ: 15 s ↓↓ 4 min ↑
							8 min ↑↑ 12 min ↓
							16 min ↓
Krcmar et al. (2021)	Volleyball players: 7 Track and field athletes: 2 Handball: 1 Soccer player: 1 CrossFit athletes:3	14F	21.9 ± 2.3	Parallel back squat	3/4/85% 1RM	5, 10 min	CMJ: 5 min ↑↑
							10 min↑
							SJ: 5 min ↑↑
Moir et al. (2011)	Volleyball players	11F	19.3 ± 0.5	Squat	HL: 1/2/50% 1RM, 1/1/70% 1RM, 1/3/90% 1RM	2 min	CMJ: ND
Crum et al. (2012)	Trained men	20M	22.1 ± 4.0	Back squat	50% 1RM back squat: 1/1/30% 1RM, 1/1/40% 1RM, 3/1/50% 1RM	30 s and 3, 5, 10, 15 min	VJ: ND
					65% 1RM back squat: 1/1/30% 1RM, 1/1/40% 1RM, 3/1/65% 1RM		
Weber et al. (2008)	Track and field athletes	12M	20.3 ± 1.7	Back squat	1/5/85% 1RM	3 min	SJ: ND
Hirayama (2014)	College weightlifters	14M	19.9 ± 1.4	Squat	1/20% 1RM+ 1/40% 1RM+ 1/60% 1RM+ 1/80% 1RM+ maximal isometric squat	3 min after each squat	VJ: 20% ↓ 40% ↑
							60% ↑↑ 80% ↑↑
							MI ↑↑
Rixon et al. (2007)	Men and female	15M, 15F	M: 23.1 ± 2.4	Dynamic squat and maximal isometric squat	1/4–6/90% ± 1.4% 3RM	3 min	CMJ: men ↑ women ↓ experienced ↑ inexperienced ND
			F: 23.4 ± 3.1				
Sharma et al. (2018)	Soccer players	7M	18.57 ± 0.94	Half squat	1/10/90% 1RM	1, 10 min	CMJ: 1 min ↓
							10 min ↑
Atalag et al. (2021)	Physically active individuals	11M, 8F	28.42 ± 7.79	Back squat	2/3/90%1RM	8min	VJ: ND
Escobar Hincapié et al. (2021)	Healthy individuals with strength training experience	12M, 5F	25 ± 1.6	Squat	3/3/0.59 m/s	5min	CMJ↓
Yi et al. (2022)	Amateur boxers	NA	19.20 ± 1.55	Squat	3/5/80%1RM squats	3, 6, 9 and 12min	CMJ: ND

Notes: ND, no significant difference; ↑, increment in jump height; ↑↑, significant increment in jump height; ↓, decrement in jump height; ↓↓, significant decrement in jump height.

Abbreviations: CA, conditioning activity; M, male; F, female; LL, low-volume and low-intensity; LH, low-volume and high-intensity; HH, high-volume and high-intensity; LV, low volume; MV, moderate volume; HV, high volume; HL, high-load; HV, high-volume.

### Risk of bias

**FIGURE 2:**
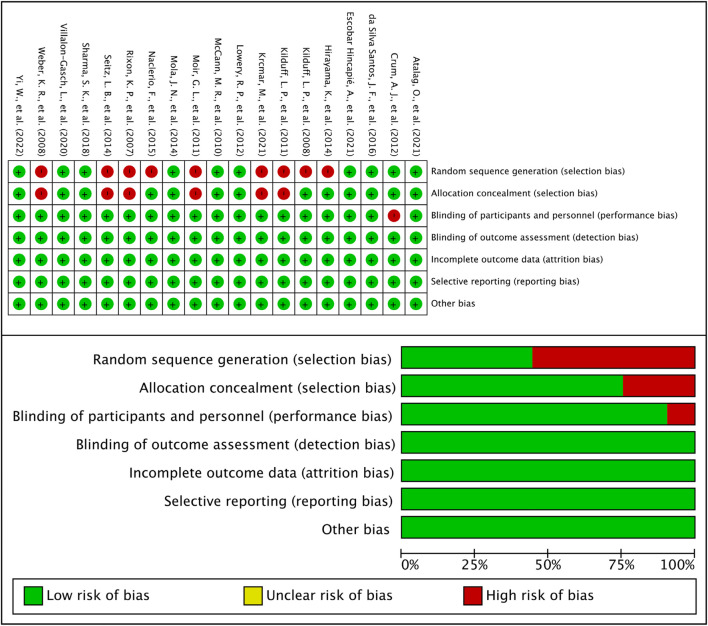
Results of Cochrane risk of bias tool.

**FIGURE 3:**
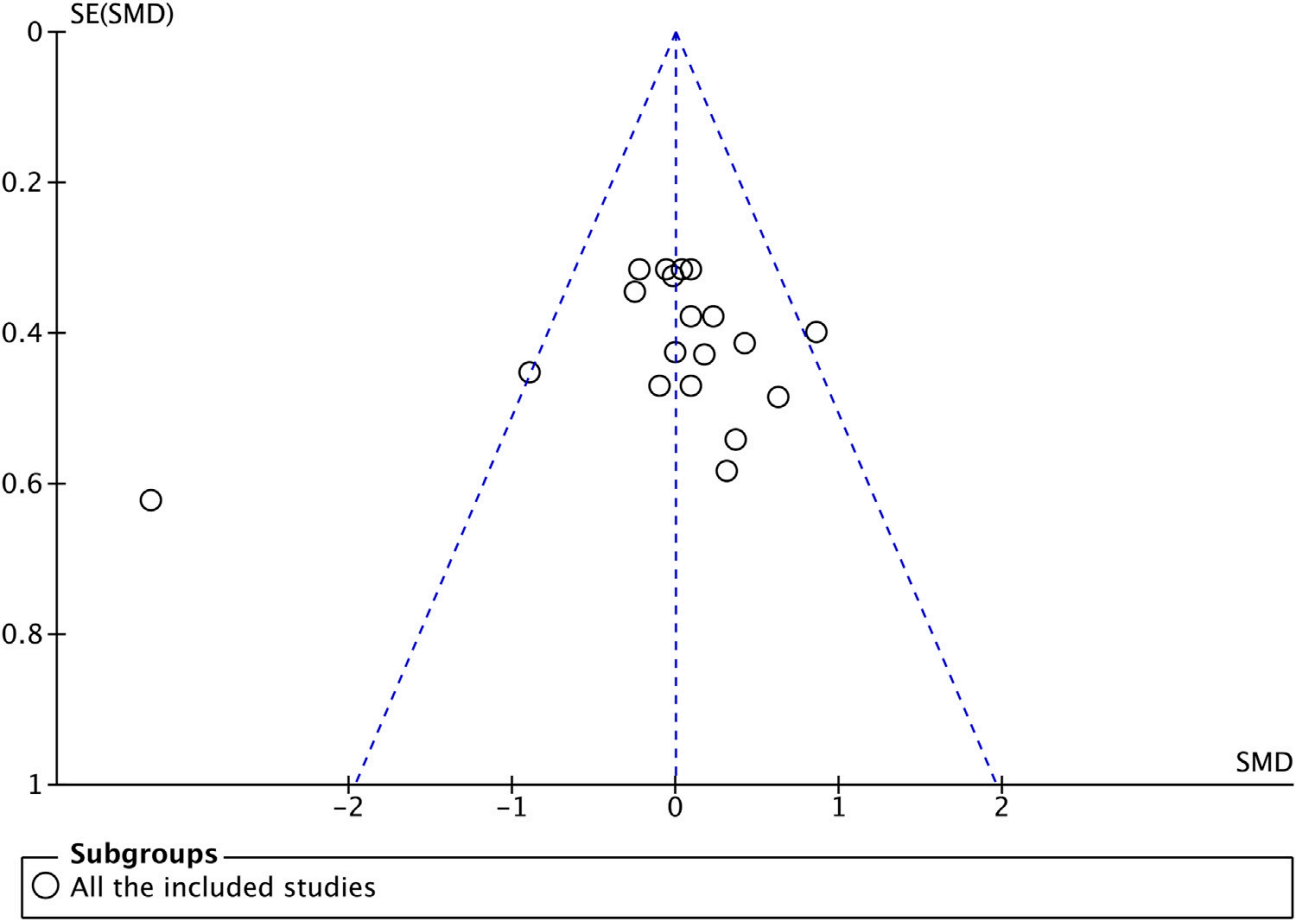
Funnel plot.

Cochrane risk assessment tool was used to evaluate the methodological quality of the included literature. The quality of the included literature was divided into three levels from high to low: high quality, medium quality, and low quality, and the result showed reasonably (Figure 2). Since the included experiments were interventional experiments and most of the articles were not RCTs, we concluded that these articles were high-risk. Moreover, the included literature also did not mention whether blinding was used, but all articles gave complete data and analysis, so we judged them to be low-risk. Publication bias was assessed visually by inspecting the funnel plot (Figure 3), publication bias was not observed from the distribution point of view, so subsequent analysis of the data will be performed.

### Main effect

Overall, 19 studies reported 105 effect sizes for meta-analysis. After analyzing the data of all included studies, we found that BS have no significant effect on overall jumping performance [Cohen’s *d* = 0.09, 95% confidence interval (CI), −0.01 to 0.18, *p* = 0.08, *I* ^2^ = 8.39%, Table 2]. To better investigate the effects of rest intervals and training intensity on jumping performance, subgroup analyses were subsequently performed.

**TABLE 2:** Results of moderator analysis.

Moderator	Cohen’s *d* (95%CI)	*I* ^2^ (%)	*p*-value
Overall	0.09 (−0.01, 0.18)	8.39	0.08
Rest interval			
0–1 min	−0.33 (−0.54, −0.13)	32.61	<0.01
2–3 min	0.15 (−0.03, 0.32)	2.18	0.10
4–7 min	0.31 (0.11, 0.51)	6.38	<0.01
8–9 min	0.26 (0.04, 0.47)	4.35	0.02
10–11 min	0.14 (−0.15, 0.44)	2.21	0.33
12–15 min	−0.03 (−0.25, 0.19)	4.12	0.79
16 min and above	0.03 (−0.30, 0.36)	3.69	0.87
Intensity			
Low-intensity	−0.00 (−0.20, 0.20)	5.50	0.97
Moderate-intensity	0.10 (−0.07, 0.27)	0.40	0.26
High-intensity	0.12 (−0.02, 0.26)	0.68	0.09
Rest interval at different intensities			
Low-intensity			
0–1 min	−0.31 (−0.80, 0.18)	1.71	0.20
2–3 min	0.10 (−0.20, 0.41)	3.74	0.48
4–7 min	0.00 (−0.46,0.46)	1.58	1.00
8–9 min	−0.05 (−0.67, 0.58)	8.33	0.88
10–11 min	−0.03 (−0.48, 0.43)	2.76	0.91
12–15 min	0.09 (−0.54, 0.72)	6.18	0.76
16 min and above	—	—	—
Moderate-intensity			
0–1 min	−0.09 (−0.33, 0.15)	55.05	0.46
2–3 min	0.24 (−0.02, 0.50)	2.32	0.07
4–7 min	0.32 (−0.02, 0.66)	8.56	0.06
8–9 min	0.34 (−0.05, 0.73)	0.89	0.08
10–11 min	0.00 (−0.64, 0.64)	5.83	1.00
12–15 min	−0.04 (−0.34, 0.25)	3.76	0.76
16 min and above	—	—	—
High-intensity			
0–1 min	−0.87 (−1.30, −0.43)	27.96	<0.01
2–3 min	0.16 (−0.16, 0.48)	2.87	0.33
4–7 min	0.41 (0.10, 0.73)	2.49	0.01
8–9 min	0.34 (0.01, 0.68)	8.66	0.04
10–11 min	0.35 (−0.17, 0.87)	2.91	0.19
12–15 min	−0.09 (−0.50, 0.33)	3.22	0.68
16 min and above	0.02 (−0.37, 0.41)	3.69	0.92

Abbreviations: CI, confidence interval.

### Subgroup analysis

Different results were shown when considering rest interval between conditioning and main activity (Table 2). Specifically, rest interval between 0–1 min was detrimental to jumping performance (Cohen’s *d* = −0.33, 95% CI, −0.54 to −0.13, *p* < 0.01, *I* ^2^ = 32.61%). Conversely, rest intervals between 4–7 min (Cohen’s *d* = 0.31, 95% CI, 0.11 to 0.51, *p* < 0.01, *I* ^2^ = 6.38%) and 8–9 min (Cohen’s *d* = 0.26, 95% CI, 0.04 to 0.47, *p* = 0.02, *I* ^2^ = 4.35%) provided favorable jumping performance outcomes. However, rest interval between 2–3 min (Cohen’s *d* = 0.15, 95% CI, −0.03 to 0.32, *p* = 0.10, *I* ^2^ = 2.18%), 10–11 min (Cohen’s *d* = 0.14, 95% CI, −0.15 to 0.44, *p* = 0.33, *I* ^2^ = 2.21%), 12–15 min (Cohen’s *d* = −0.03, 95% CI, −0.25 to 0.19, *p* = 0.79, *I* ^2^ = 4.12%), and 16 min and above (Cohen’s *d* = 0.03, 95% CI, −0.30 to 0.36, *p* = 0.87, *I* ^2^ = 3.69%) had no significant effect on jumping performance.

In addition, different results were shown when considering the training intensity of BS (Table 2). Included studies were divided into 3 subgroups based on % 1RM (low-intensity, ≤60% 1RM; moderate-intensity, 60%–85% 1RM; high-intensity, >85% 1RM) (Wilson et al., 2013). Our results showed that training intensity had no significant effect on jump performance (low-intensity, Cohen’s *d* = −0.00, 95% CI, −0.20 to 0.20, *p* = 0.97, *I* ^2^ = 5.50%; moderate-intensity, Cohen’s *d* = 0.10, 95% CI, −0.07 to 0.27, *p* = 0.26, *I* ^2^ = 0.40%; high-intensity, Cohen’s *d* = 0.12, 95% CI, −0.02 to 0.26, *p* = 0.09, *I* ^2^ = 0.68%).

We next determined whether rest intervals at different intensities would affect jumping performance (Table 2). We found that rest intervals at low-intensity [0–1 min (Cohen’s *d* = −0.31, 95% CI, −0.80 to 0.18, *p* = 0.20, *I* ^2^ = 1.71%), 2–3 min (Cohen’s *d* = 0.10, 95% CI, −0.20 to 0.41, *p* = 0.48, *I* ^2^ = 3.74%), 4–7 min (Cohen’s *d* = 0.00, 95% CI, −0.46 to 0.46, *p* = 1.00, *I* ^2^ = 1.58%), 8–9 min (Cohen’s *d* = −0.05, 95% CI, −0.67 to 0.58, *p* = 0.88, *I* ^2^ = 8.33%), 10–11 min (Cohen’s *d* = −0.03, 95% CI, −0.48 to 0.43, *p* = 0.91, *I* ^2^ = 2.76%) and 12–15 min (Cohen’s *d* = 0.09, 95% CI, −0.54 to 0.72, *p* = 0.76, *I* ^2^ = 6.18%)] and moderate-intensity [0–1 min (Cohen’s *d* = −0.09, 95% CI, −0.33 to 0.15, *p* = 0.46, *I* ^2^ = 55.05%), 2–3 min (Cohen’s *d* = 0.24, 95% CI, −0.02 to 0.50, *p* = 0.07, *I* ^2^ = 2.32%), 4–7 min (Cohen’s *d* = 0.32, 95% CI, −0.02 to 0.66, *p* = 0.06, *I* ^2^ = 8.56%), 8–9 min (Cohen’s *d* = 0.34, 95% CI, −0.05 to 0.73, *p* = 0.08, *I* ^2^ = 0.89%), 10–11 min (Cohen’s *d* = 0.00, 95% CI, −0.64 to 0.64, *p* = 1.00, *I* ^2^ = 5.83%) and 12–15 min (Cohen’s *d* = −0.04, 95% CI, −0.34 to 0.25, *p* = 0.76, *I* ^2^ = 3.76%)] had no significant effect on jumping performance. However, at high-intensity, rest intervals between 0–1 min was detrimental to jumping performance (Cohen’s *d* = −0.87, 95% CI, −1.30 to −0.43, *p* < 0.01, *I* ^2^ = 27.96%), rest intervals between 4–7 min (Cohen’s *d* = 0.41, 95% CI, 0.10 to 0.73, *p* = 0.01, *I* ^2^ = 2.49%) and 8–9 min (Cohen’s *d* = 0.34, 95% CI, 0.00 to 0.68, *p* = 0.04, *I* ^2^ = 8.66%) provided favorable jumping performance outcomes, and rest intervals between 2–3 min (Cohen’s *d* = 0.16, 95% CI, −0.16 to 0.48, *p* = 0.33, *I* ^2^ = 2.87%), 10–11 min (Cohen’s *d* = 0.35, 95% CI, −0.17 to 0.87, *p* = 0.19, *I* ^2^ = 2.91%), 12–15 min (Cohen’s *d* = −0.09, 95% CI, −0.50 to 0.33, *p* = 0.68, *I* ^2^ = 3.22%), and 16 min and above (Cohen’s *d* = 0.02, 95% CI, −0.37 to 0.41, *p* = 0.92, *I* ^2^ = 3.69%) had no significant effect on jumping performance.

## Discussion

The aim of this study was to explore the effects of rest intervals and training intensity on jumping performance due to PAPE. From 2518 search records initially identified, 19 studies were considered eligible for meta-analysis. Our meta-analysis results showed that BS had no significant effect on improving jumping performance due to PAPE. Subgroup analysis showed that rest interval between 0–1 min was detrimental to jumping performance. Conversely, rest intervals between 4–7 and 8–9 min provided favorable jumping performance outcomes. However, rest intervals between 2–3, 12–15, and 16 min and above and training intensity had no significant effect on jumping performance. In addition, when considering rest intervals at different intensities, we found that all rest intervals at low-intensity and moderate-intensity had no significant effect on jumping performance. However, at high-intensity, rest intervals between 0–1 min was detrimental to jumping performance, rest intervals between 4–7 and 8–9 min provided favorable jumping performance outcomes, and rest intervals between 2–3, 10–11, 12–15, and 16 min and above had no significant effect on jumping performance, which was consistent with the results of rest interval.

Following a conditioning activity protocol, two states of muscle fatigue and potentiation coexist (Boullosa et al., 2011; Latorre-Román et al., 2014), and the net balance between fatigue and potentiation may influence the subsequent performance. Previous studies have shown that elevated muscle temperature may lead to potentiation, mainly due to intense exercise caused by increased arterial blood flow and muscle metabolism, and thus the time required for vascular bed dilation and muscle perfusion may explain the delay in temperature increase and potentiation after contraction (Chaouachi et al., 2011; Cochrane et al., 2010; McGowan et al., 2015). The rest interval used may determine which is superior, fatigue or potentiation, and whether enhanced performance is achieved at different rest intervals. Previous studies have shown that fatigue may dominate potentiation during the early recovery phase after conditioning activities (Beato et al., 2020; Duncan et al., 2014; Tillin and Bishop, 2009), and as shown in the present study, rest interval between 0–1 min showed a significant decrease in jumping performance. If the rest interval is too short, fatigue may outweigh the potentiation effect, and conversely, the optimal potentiation effect may disappear, which has no effect on performance (Masiulis et al., 2018). Thus, the height of the muscle force-time curve increases and then decreases during the performance. Therefore, the rest interval plays a decisive role in the following jumping performance.

Our meta-analysis results showed that BS had no significant effect on improving jumping performance due to PAPE, we suspected that this may be related to a decrease in jumping performance during the fatigue phase, so we conducted subgroup analyses to interpret the results. The results of previous studies on the optimal rest interval for PAPE effects remain inconsistent, probably due to the long rest intervals, which were divided into 4 groups: less than 2, 3–7, 7–10 min, and more than 10 min (Gouvea et al., 2013; Wilson et al., 2013; Dobbs et al., 2019). Based on the information provided by the included studies, we divided the rest intervals into 7 periods: 0–1, 2–3, 4–7, 8–9, 10–11, 12–15, and 16 min and above to explore whether the PAPE effects changed after the rest interval refinement. Our results showed that a rest interval of 4–9 min had a beneficial impact on jump height, and an interval range of 4–7 min seems to be the best rest interval between conditioning activity and jumping performance. The appearance of optimal jumping performance after pre-stimulation showed delayed characteristics due to the fatigue effects, with significant differences between individuals (Tillin and Bishop, 2009). Previous meta-analyses have found different results. For example, Gouvea et al. (2013) considered 8–12 min as the optimal rest interval, while Dobbs et al. (2019) showed better jumping performance at 3–7 min. The reason for the difference between the present study and previous meta-analyses may be that studies included in the present study used BS as a means of inducing PAPE, but previous studies were not limited to BS, which may lead to a high degree of heterogeneity. Thus, our study provided additional evidence for the choice of rest intervals after BS intervention. Although the mechanism of PAPE had not been fully revealed, it was hypothesized that the beneficial effect of PAPE on jumping performance may be enhanced by the following mechanisms: (1) pre-contraction stimulation enhances the muscle myosin-regulated light chain phosphorylation (Sweeney et al., 1993); (2) after pre-contraction stimulation, increased neural activity leads to an increase in motor units controlled by motor neurons (Cazas et al., 2013); and (3) the rotation angle of muscle fibers contracts due to pre-contraction stimulation (Mahlfeld et al., 2004). A previous study showed that the potentiation is most effective at low myoplasm Ca^2+^ concentrations (Smith, 2014) and is therefore more likely to occur in twitch contractions or low-frequency tetanic contractions with moderate- or high-intensity, but not at high stimulation frequency with low intensity, which will produce saturated levels of Ca^2+^, thus preventing any effects of increased Ca^2+^ sensitivity. In addition, another study has confirmed that the potentiation effect is mainly attributed to an increase in muscle temperature, where an increase in temperature has different effects on Ca^2+^ sensitivity, but an increase in temperature is generally associated with a decrease in Ca^2+^ sensitivity. This reduces the likelihood of myosin binding to actin in the presence of Ca^2+^ (Lee et al., 1991). Increased muscle temperature does correlate with an increase in the rate of strength development and a shortening of velocity (Gossen et al., 2001), which can trigger significant performance enhancements in activities requiring high levels of muscular power output, such as vertical jumping (Kavvoura et al., 2018).

Considering the inconsistency in defining rest intervals, we should also consider other factors. The wide range of intervals is most likely related to individual fatigue and recovery. The potentiation response relies on the stimulation applied to the muscle or motor nerve during the conditioning contraction. Stimulation is closely related to the intensity of the conditioning activity, which is also an important issue for practitioners to be aware of. One study showed that a 1°C increase in muscle temperature induced by passive warming (water immersion) increased maximal power by 5.1% in handgrip exercise (Binkhorst et al., 1977). Intense exercise leads to increased arterial inflow and muscle metabolism, which resulted in peak temperature increases a few minutes after exercise commencement (Gonzalez-Alonso et al., 2000). Nonetheless, moderate- and high-intensity exercise can achieve greater temperature increases, but not low-intensity exercise (Zois et al., 2011; Neiva et al., 2014; West et al., 2016). Improvements in vertical jump were found at 4, 8, 12, 16, and 20 min after the protocol, showing that a single set of high-intensity flywheel resistance training led to PAPE in CMJ (Maroto-Izquierdo et al., 2020). Lowery et al. (2012) performed 3 different intensity protocols in well-trained subjects and concluded that moderate- and high-intensity loads may benefit the following explosive performance, and high-intensity loads may prolong the duration of PAPE. Meanwhile, most studies have implemented low-intensity warm-up exercises that are insufficient to achieve full muscle temperature elevation and performance potentiation (Evetovich et al., 2015; Wallace et al., 2019), which was inconsistent with our results. Therefore, moderate-intensity exercise was highly recommended in the previous studies (Chaouachi et al., 2011; Crum et al., 2012; Lowery et al., 2012; Naclerio et al., 2015; Sotiropoulos et al., 2010; Vanderka et al., 2016). However, Fountas et al. (2012) found opposite results after a high-intensity setting in recreational individuals. These inconsistent results also reflect the need for good monitoring of the rest interval during the implementation of the moderate- or high-intensity induction method. Therefore, we determined whether training intensity would affect PAPE effects. The results of this study showed no significant effect of training intensity on jumping performance, which was inconsistent with previous studies, showing that moderate-intensity and high-intensity conditioning activity induced a PAPE effect and significantly improved jumping performance, whereas low-intensity conditioning activity had no significant effect on jumping performance (Berning et al., 2010; Borba et al., 2017; Sale, 2002; Smilios et al., 2010). This may be due to the fact that all rest intervals were analyzed in the study, where rest interval between 0–1 min was detrimental to jump performance and rest intervals between 2–3, 12–15, and 16 min and above had no significant effect on jumping performance. When all rest intervals were analyzed together, it was possible to attenuate the effect of training intensity on jumping performance due to PAPE. Therefore, we next determined whether rest intervals at different intensities would affect jumping performance.

When considering rest intervals at different intensities, we found that all rest intervals at low-intensity and moderate-intensity had no significant effect on jumping performance. However, at high-intensity, rest intervals between 0–1 min was detrimental to jumping performance, rest intervals between 4–7 and 8–9 min provided favorable jumping performance outcomes, and rest intervals between 2–3, 10–11, 12–15, and 16 min and above had no significant effect on jumping performance, which was consistent with the results of rest interval and previous studies (Fukutani et al., 2014; Zois et al., 2013). Therefore, we recommended to use high-intensity BS to induce PAPE in future studies, with an optional rest interval of 4–7 min.

Some potential limitations of this meta-analysis should be acknowledged. First, most of the samples were experienced subjects, and there were fewer inexperienced subjects, and most of them were males, so we did not perform subgroup analysis on gender and training experience. Second, other influencing factors of PAPE, such as group number, volume, and frequency, were not discussed in the study due to the small number of studies. Finally, the intervention in this study was limited to BS, so the results could not be replicated with other methods. Therefore, future studies were needed to conduct more in-depth studies based on the above shortcomings.

## Conclusions

Our study indicated that both low-intensity and moderate-intensity BS failed to induce PAPE, and it is recommended that future studies use high-intensity BS to induce PAPE. A rest interval of 4–9 min had a beneficial impact on jump height, and an interval range of 4–7 min seems to be the best rest interval between conditioning activity and jumping performance.

## Data Availability

The original contributions presented in the study are included in the article/supplementary material, further inquiries can be directed to the corresponding authors.
